# Alterations via inter-regional connective relationships in Alzheimer’s disease

**DOI:** 10.3389/fnhum.2023.1276994

**Published:** 2023-11-09

**Authors:** Xiaomei Ren, Bowen Dong, Ying Luan, Ye Wu, Yunzhi Huang, the Alzheimer's Disease Neuroimaging Initiative

**Affiliations:** ^1^College of Electrical Engineering, Sichuan University, Chengdu, China; ^2^Department of Radiology, Zhongda Hospital, School of Medicine, Southeast University, Nanjing, China; ^3^School of Computer Science and Engineering, Nanjing University of Science and Technology, Nanjing, China; ^4^Institute for AI in Medicine, School of Artificial Intelligence (School of Future Technology), Nanjing University of Information Science and Technology, Nanjing, China

**Keywords:** cortical thickness, cortical covariance network, vortex-wise general linear model, seed-based functional connectivity, group-level independent component analysis

## Abstract

Disruptions in the inter-regional connective correlation within the brain are believed to contribute to memory impairment. To detect these corresponding correlation networks in Alzheimer’s disease (AD), we conducted three types of inter-regional correlation analysis, including structural covariance, functional connectivity and group-level independent component analysis (group-ICA). The analyzed data were obtained from the Alzheimer’s Disease Neuroimaging Initiative, comprising 52 cognitively normal (CN) participants without subjective memory concerns, 52 individuals with late mild cognitive impairment (LMCI) and 52 patients with AD. We firstly performed vertex-wise cortical thickness analysis to identify brain regions with cortical thinning in AD and LMCI patients using structural MRI data. These regions served as seeds to construct both structural covariance networks and functional connectivity networks for each subject. Additionally, group-ICA was performed on the functional data to identify intrinsic brain networks at the cohort level. Through a comparison of the structural covariance and functional connectivity networks with ICA networks, we identified several inter-regional correlation networks that consistently exhibited abnormal connectivity patterns among AD and LMCI patients. Our findings suggest that reduced inter-regional connectivity is predominantly observed within a subnetwork of the default mode network, which includes the posterior cingulate and precuneus regions, in both AD and LMCI patients. This disruption of connectivity between key nodes within the default mode network provides evidence supporting the hypothesis that impairments in brain networks may contribute to memory deficits in AD and LMCI.

## 1. Introduction

Alzheimer’s disease (AD), a specific form of dementia, is a neurodegenerative disorder characterized by the presence of tau and β-amyloid deposition as well as neuronal loss. Alterations in inter-regional correlation networks within the brain are believed to contribute to atrophy and neurodegeneration in associated brain regions, ultimately leading to cognitive decline (Vemuri and Jack, 2010; Yu et al., 2021). Mild cognitive impairment (MCI) refers to a mild cognitive decline that does not interfere with daily activities. More than half of MCI cases develop dementia within 5 years (Arbabshirani et al., 2017). Investigating abnormalities in inter-regional covariant and connective correlation in AD and MCI is pivotal for early detection of AD.

Magnetic resonance imaging (MRI) is a promising imaging modality due to its non-invasive detection, accessibility, and non-ionizing radiation. Previous studies using T1-weighted structural MRI (sMRI) have demonstrated reduced cortical thickness (CT) in specific cortical regions across various conditions (Canu et al., 2017; Park et al., 2017; Phillips et al., 2019). However, such measures of cortical atrophy fail to capture inter-regional connectivity within the brain. In contrast, metrics assessing inter-regional correlations have been shown to be superior in detecting Aβ pathology and Alzheimer’s disease progression (Neitzel et al., 2019) compared to measures of brain atrophy. The cortical covariance analysis examines the CT covariant relationship across brain regions, and the functional connectivity (FC) measures inter-regional synchronous correlation using Pearson’s correlation of blood oxygen level dependent (BOLD) sequences from resting-state MRI (rsfMRI). Recent studies have revealed alterations in inter-regional correlation under different conditions, including structural covariance (Tuladhar et al., 2015; Nestor et al., 2017; Valk et al., 2017; Phillips et al., 2019) and FC networks (Canu et al., 2017; Yan et al., 2017; Kuang et al., 2019; Berron et al., 2020; Smith et al., 2021). These studies used pre-selected region of interests (ROIs) based on specific atlases (Nestor et al., 2017; Phillips et al., 2019) or meta-analytical results (Valk et al., 2017) for correlation analysis between brain regions. The ROI-based analysis relies solely on selected ROIs and may be more sensitive to variable atrophied areas (Smitha et al., 2017). The voxel-wise method automatically defines ROIs boundaries based on data, providing more precise anatomical location of brain atrophy (Phillips et al., 2019). In this study, we conducted the vertex-wise CT analysis at the whole brain level to identify the significant different regions in AD and LMCI compared to the cognitively normal (CN). These regions were used as seed ROIs for inter-regional structural covariant and functional connectivity analysis.

Besides FC analysis (Smitha et al., 2017; Delli Pizzi et al., 2019; Neitzel et al., 2019; Zhao et al., 2019; Berron et al., 2020; Smith et al., 2021), rsfMRI has been widely used in group independent component analysis (group-ICA). This data-driven method aggregates all voxels of all subjects by temporally concatenating BOLD sequences to investigate voxel-to-voxel interaction and isolates distinct spatially independent maps known as group-ICA networks (Smitha et al., 2017; Dadi et al., 2019). Group-ICA is advantageous due to its prior independence and noise insensitivity, and has successfully identified default mode network (DMN) in patients with AD (Park et al., 2017; Smitha et al., 2017; Son et al., 2022). This motivates us to employ group-ICA to identify significant differences in ICA networks between patients and CN.

Numerous studies have reported improved brain networks results by jointly using structural and functional MRI, as well as ICA networks (Canu et al., 2017; Park et al., 2017; Smitha et al., 2017; Beheshtian et al., 2021; Caspers et al., 2021; Son et al., 2022). However, the consistency among cortical covariance, functional connectivity and ICA networks in AD has not yet been investigated. We hypothesized that brain regions belonging to subnetworks of typical brain networks would exhibit consistent changes across multiple correlation modalities in AD. Specifically, we sought to identify brain regions that consistently emerged in the three types of networks (cortical covariance, functional connectivity, group-ICA). Regions meeting this consistency criteria were defined as AD-specific inter-regional correlation networks.

## 2. Materials and methods

**Figure 1:**
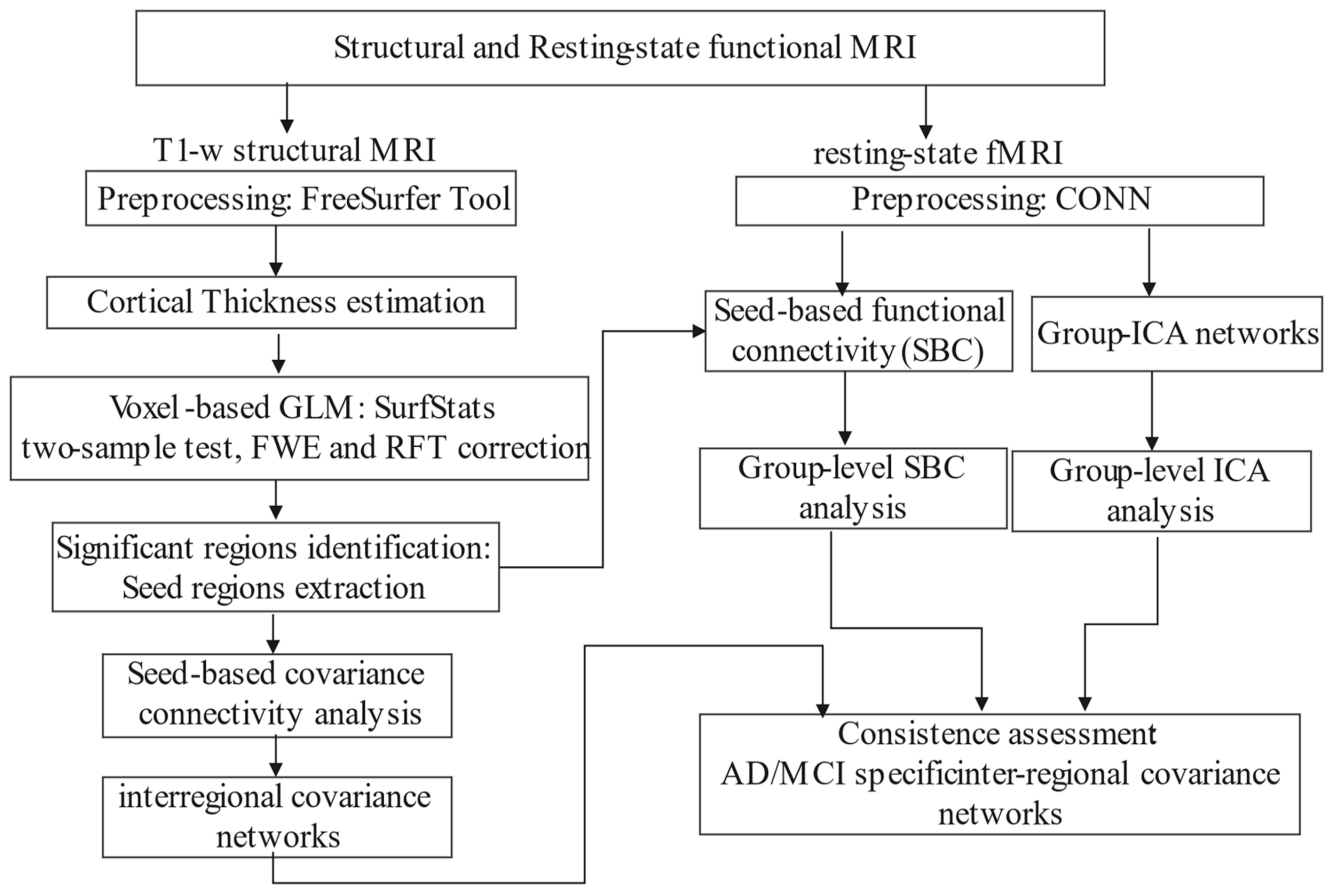
Schematic diagram of our study.

Our two-branch analytical pipeline is illustrated in Figure 1, with one branch dedicated to structural covariance analysis using sMRI data and the other for FC analysis using rsfMRI data. After independently constructing the structural and functional correlation networks, we distill the consistent elements between them as distinct inter-regional connectivity for distinguishing AD or MCI from CN.

### 2.1. Data

Data used in this study were from the Alzheimer’s Disease Neuroimaging Initiative (ADNI) database.^1^ The ADNI was launched in 2003 as a public-private partnership functional network led by Principal Investigator Michael W. Weiner, MD. The primary goal of ADNI has been to test whether serial MRI, positron emission tomography (PET), other biological markers, and clinical and neuropsychological assessment can be combined to measure the progression of MCI and early AD.

### 2.2. Study participants

**Figure 2:**
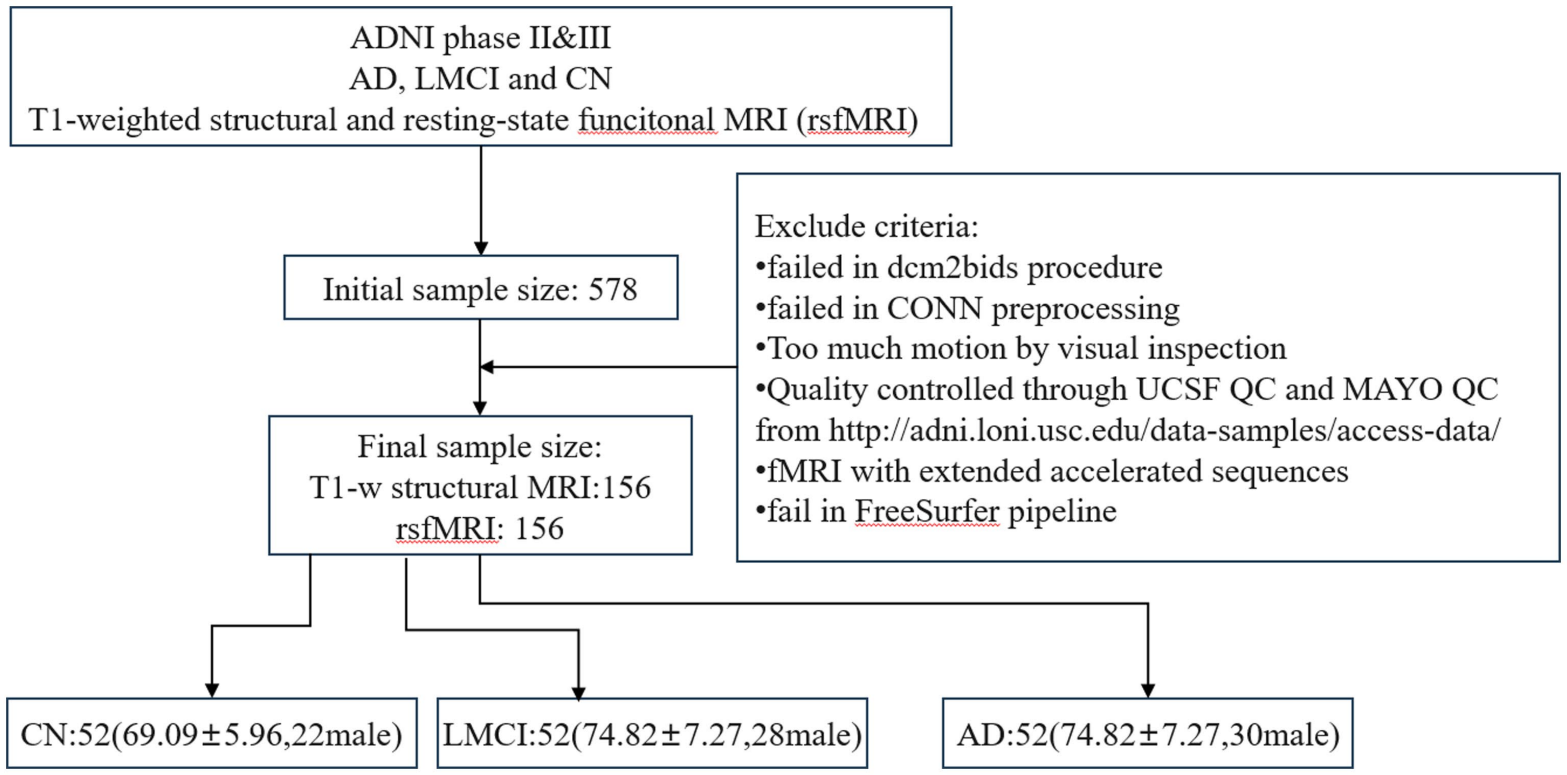
The workflow of selecting participants.

The study included records from the ADNI2 and ADNI3 phases, which were acquired using 3 T scanners (Siemens, Philips and GE). All data underwent quality control based on UCSF QC outcomes and MAYO QC levels from LONI. More detailed documents can be found in the ADNI dataset. For rsfMRI, sequences with multiband echo planar imaging (EPI) acquisition and data with excessive motion artifacts or scrubbling values greater than 25 during CONN preprocessing were excluded. For sMRI, subjects who failed to pass the FreeSurfer pipeline were also excluded. Finally, to maintain a balanced sample size, three diagnostic groups (AD/LMCI/CN: 52/52/52) were enrolled. In our study, the original downloaded sample size was 578 participants, and after applying selection criteria we ended up with a final sample size of 156 as shown in Figure 2.

### 2.3. Image acquisition

All MRI scans in our study were collected exclusively during the Baseline Visit periods, following the participants determination of eligibility and completion of the informed consent process. The T1w sMRI data were acquired with accelerated sagittal magnetization-prepared rapid gradient echo sequence (in-plane resolution = 1.0 × 1.0 mm^2^; slice thickness = 1 mm; repetition time = 2,300 ms; echo time = 3.0 ms; flip angle = 9°; field of view: 224 mm). The axial rsfMRI data were acquired with EPI BOLD sequence (pixel spacing x = 3.4 mm; pixel spacing y = 3.4 mm; slice thickness = 3.4 mm; matrix: 64 × 64; flip angle = 90°; slices = 197; echo time = 30.0 ms; repetition time = 3 s; field of view: 212 mm).

### 2.4. Data preprocessing

The structural and rsfMRI data in DICOM format were downloaded from LONI and converted to NifTi files using dcm2niix and Dcm2Bids tools (Gorgolewski et al., 2016). Data that failed to pass Dcm2Bids procedure were excluded.

#### 2.4.1. T1w structural data

The CT data were estimated through reconstruction of cortical surface from the T1w image using the default FreeSurfer pipeline (Dale et al., 1999). Our analysis began with brain segmentation of white matter (WM), grey matter (GM) and cerebrospinal fluid (CSF), followed by surface reconstruction. We generated the WM and pial surface, performed surface inflation and spherical flattening transformations, and assigned a Destrieux atlas label to each cortical voxel using cortical parcellation (Fischl et al., 2004). Statistical results of cortical parcellation were then created, where T1w images were registered to the surface template and smoothed with a 10 mm full-width half-maximum (FWHM) Gaussian kernel. Finally, the cortical thickness (CT) of each cortex was estimated as the shortest distance between the WM boundary and the pial surface (Fischl and Dale, 2000).

#### 2.4.2. Resting state functional data

The preprocessing results of rsfMRI data come from analyses performed using CONN (Whitfield-Gabrieli and Nieto-Castanon, 2012) release 22.a (Nieto-Castanon and Whitfield-Gabrieli, 2022) and SPM (Penny et al., 2011) release 12.6470.

After removing the first 4 volumes, rsfMRI data were preprocessed using a default preprocessing pipeline (Nieto-Castanon, 2020). In brief, functional data were firstly realigned (Friston et al., 1995; Andersson et al., 2001) and resampled to correct for motion. Potential outlier scans were then identified (Whitfield-Gabrieli et al., 2011). Functional and anatomical data were normalized into standard MNI space, segmented into GM, WM, and CSF tissue, and resampled to 2 mm isotropic voxels (Ashburner and Friston, 2005; Ashburner, 2007; Calhoun et al., 2017). Next, functional data were smoothed with a Gaussian kernel of 6 mm FWHM. Last, functional data were denoised using a standard denoising pipeline (Whitfield-Gabrieli and Nieto-Castanon, 2012) including the regression of potential confounding effects characterized by WM timeseries, CSF timeseries, motion parameters (Friston et al., 1996), outlier scans (Power et al., 2014), and linear trends, followed by bandpass filtering (Hallquist et al., 2013) between 0.001 and 0.2 Hz.

FC values associated with a specific seed were estimated while controlling for all other seeds. The strength of FC was represented by Fisher-transform of semi-partial correlation coefficients from a weighted general linear model (GLM) (Nieto-Castanon, 2020). This approach allowed for modeling the association of BOLD time-series between each seed and other voxel in the brain.

#### 2.4.3. Group-level ICA networks

Group-ICA (Calhoun et al., 2001) were performed to estimate 40 temporally coherent networks from the rsfMRI data across all subjects. The BOLD time series from every voxel in the brain across all subjects were temporally concatenated. For each subject, a singular value decomposition of the *z*-score normalized BOLD signal with 64 components was separately used as a subject-specific dimensionality reduction step. The dimensionality was further reduced to 40 components and a fast-ICA fixed-point algorithm (Hyvarinen, 1999) was used to identify spatially independent group-level networks. Lastly, a back-projection (Erhardt et al., 2011) was used to compute ICA networks separately for each subject.

### 2.5. Statistical analysis

#### 2.5.1. Demographic and volumetric data analysis

We evaluated the normal distribution of continuous demographic variables (age and years of education), average CT and the total GM volume (GMV) using a single-sample Kolmogorov–Smirnov test. Group-wise differences were assessed using a two-sample Kolmogorov–Smirnov test. The analysis of sex and type of scanner were conducted using the Mann–Whitney rank sum test. All statistical analyses were performed on the Matlab platform.

#### 2.5.2. Voxel-wise cortical thickness analysis

The vertex-wise GLM was conducted using the SurfStat Toolbox^2^ for CT analysis. The CT value at each vertex was modeled as the dependent variable, with group (CN, AD, LMCI) as the independent variable. Age, sex, education, scanner type, and total GMV were included as covariates. Cluster-level statistics were thresholded at *p* < 0.001 for AD vs. CN and *p* < 0.025 for LMCI vs. CN to correct for multiple comparisons using random field theory (RFT). A cluster extent threshold of *p* < 0.05 family-wise error (FWE) rate was then applied to control the probability of reporting false positive clusters. The cortical regions demonstrating significant group differences were mapped onto the Destrieux atlas to generate ROIs called seeds. These regions were also mapped onto the Anatomical Automatic Labeling (AAL) atlas in MNI space to create AAL-based seeds for FC analysis.

#### 2.5.3. Cortical covariant networks

An interactive GLM approach was used to examine whether a specified seed region exhibited distinct cortical covariance patterns across the entire surface in patients compared to controls. Mathematically, the interactive GLM can be formulated as follows:


(1) $$CT_{i}=\left(\begin{array}{l} \beta_{i0}+\beta_{i1}*\mathrm{Sex}+\beta_{i2}*\mathrm{Age}\\ +\beta_{i3}*\mathrm{Diagnosis}+\beta_{i4}*\mathrm{Education}\\ +\beta_{i5}*\mathrm{Scanner}+\beta_{i6}*GMV \end{array}\right)*\left(1+\beta_{i7}*CT_{\mathrm{seed}}\right)$$

where * denotes an interaction, the weight coefficients of each covariates are denoted by β *_i_*, *CT_i_* represents the CT data of the *i*th voxel, and *CTseed* is the average CT of a specific seed, GMV refers to total GMV, Diagnosis is the group identifier. The contrast for comparing AD and CN can be: contrast = CTseed.*Diagnosis.CN-CTseed.*Diagnosis.AD. The results were corrected using RFT at a cluster threshold of *p* = 0.025, controlling probability for FWE at cluster-wise threshold *PFWE* < 0.05. All surviving regions after correction were matched onto AAL atlas.

#### 2.5.4. Group-level analysis of functional connective networks

Group-level analyses of functional networks were performed using a GLM (Nieto-Castanon, 2020). A separate GLM was estimated with first-level connectivity measures (FC connective maps/ICA maps) at this voxel as dependent variables, and group identifiers as independent variables, and covariates included age, sex, years of education and scanner type. Voxel-level hypotheses were evaluated using multivariate parametric statistics with random-effects across subjects. Inferences were performed at the level of individual cluster based on parametric statistics from Gaussian RFT (Worsley et al., 1996; Nieto-Castanon, 2020). Results were thresholded using a combination of a cluster-forming *p* < 0.0025 voxel-level threshold and a familywise corrected p-FDR < 0.05 cluster-size threshold (Chumbley et al., 2010).

#### 2.5.5. The consistent inter-regional correlative networks

The inter-regional correlation networks, estimated using the three approaches mentioned above, included brain regions that are structural covaried or functionally connected with each other or in the IC network. The regions which consistently emerged in all or any two of the three networks were identified as inter-regional connective networks.

## 3. Results

### 3.1. Study demographics

The demographic data, mean CT and the total GMV are presented in Table 1. Except for the total GMV and education years showing no significant difference (*p* < 0.05) between LMCI and CN, other variables showed significant differences (*p* > 0.05) for AD and LMCI compared with CN. Therefore, these demographic variables are used as covariates in cortex-based GLM analysis to regress out their effects.

**Table 1:** Demographic data, the mean CT and the GMV data.

	AD	LMCI	CN	AD vs. CN (value of *p*)	LMCI vs. CN (value of *p*)
Mean Age ± SD	74.82 ± 7.27	71.45 ± 7.76	69.09 ± 5.96	2.4242E-07	0.0056
Sex (male/female)	30/22	28/24	22/30	/	/
Mean Education years ± SD	15.87 ± 2.51	16.06 ± 2.65	16.92 ± 1.93	0.0373	**0.3831**
Scanner (SI/PHI/GE)	15/23/14	23/20/9	1934/6/12	/	/
Mean CT ± SD	2.3253 ± 0.0733	2.4158 ± 0.0794	2.4485 ± 0.0820	2.30E-08	0.0207
The total GMV ± SD (mm^3^)	814,230 ± 93,641	865,340 ± 80,644	905,130 ± 82,043	0.0013	**0.1711**

SI, Siemens; PHI: Philips; SD, standard deviation; AD, Alzheimer’s disease; LMCI, Late mild cognitive impairment; CN, Cognitively normal; GMV, the total grey matter volume; CT, cortical thickness.The data in bold showed the total GMV and education years with no significant difference between groups, the others are between-group different.

### 3.2. Cortical thickness and cortical covariant correlation

#### 3.2.1. Cortical thickness

Multiple regions exhibited CT thinning in AD and LMCI patients compared to controls, but no cortical thickening was found in either. Table 2 presents the regions that display cortical thinning in AD and LMCI compared to controls after being mapped to the Destrieux atlas. Patients with AD exhibited cortical thinning in bilateral entorhinal and parahippocampal, left middle temporal cortex, temporal pole, as well as inferior temporal cortex, consistent with prior previous studies (Vemuri and Jack, 2010; Phillips et al., 2019). As shown, LMCI patients exhibited less pronounced (*p* < 0.0025) cortical atrophy in the left parahippocampal and entorhinal cortex, and the right middle and posterior cingulate gyrus. The diagram corresponding to the results in Table 2 is shown in Supplementary Figure S1.

**Table 2:** The cortical thickness thinning regions in AD and LMCI.

	Destrieux atlas-based CT thinning regions			AAL atlas-based CT thinning regions		
	Atlas-based regions	Voxel number	The thinning ratio	Atlas-based regions	Voxel number	The thinning ratio
AD	L_entorhinal	656	59.53	ParaHippocampal_L	1982	25.12
	L_fusiform	70	1.48	Fusiform_L	1,147	6.26
	L_inferiortemporal	657	14.88	Temporal_Inf_L	2,682	10.46
	L_middletemporal	638	14.33	Temporal_Mid_L	1871	4.75
	L_parahippocampal	552	30.03	Temporal_Pole_Mid_L	2,477	41.39
	L_superiortemporal	69	0.95	Temporal_Pole_Sup_L	1,412	13.81
	L_temporalpole	738	87.96	Temporal_Pole_Mid_R	386	4.08
	R_entorhinal	499	55.32	ParaHippocampal_R	1761	19.51
	R_parahippocampal	523	30.02	Fusiform_R	438	2.17
LMCI	L_entorhinal	403	36.569873	ParaHippocampal_L	1,111	14.0793309
	L_parahippocampal	124	6.74646355	Fusiform_L	294	1.60366552
	R_isthmuscingulate	596	24.958124	Cingulum_Mid_R	481	2.75771127
	R_posteriorcingulate	73	2.43820975	Cingulum_Post_R	447	16.8425019
				Precuneus_R	250	0.9584787

The atrophied proportion is the ratio of the voxel number with CT different in the atlas-based region to the total number of that region.

Note that the cortical thinning regions being mapped to the AAL atlas are also shown in Table 2. In this study, ROIs from Destrieux and AAL atlases were used for cortical covariance and functional connectivity analysis, respectively.

#### 3.2.2. Cortical covariant networks

The cortical covariant networks for all seeds were derived using the method described in Section 2.5.3. Table 3 summarizes the altered cortical covariant networks in AD and LMCI compared to CN.

**Table 3:** The altered cortical covariant networks in AD and LMCI.

Contrast	Destrieux atlas-based seeds	Regions of changed cortical covariation	Number of voxel
AD<CN	L_entorhinal	Cingulum_Post_L	216
		Cingulum_Mid_R	101
		Cingulum_Post_R	500
		Precuneus_R	779
	R_entorhinal	Cingulum_Post_L	141
AD>CN	L_entorhinal	Temporal_Pole_Sup_L	2,146
		Temporal_Pole_Mid_L	1,687
	L_fusiform	ParaHippocampal_L	772
		Temporal_Pole_Sup_L	533
	L_inferiortemporal	Fusiform_L	820
		Temporal_Sup_L	288
		Temporal_Pole_Sup_L	3,924
		Temporal_Mid_L	1,422
		Temporal_Pole_Mid_L	1819
		Temporal_Inf_L	3,430
	L_middletemporal	Temporal_Pole_Sup_L	2,966
		Temporal_Pole_Mid_L	1,686
		Temporal_Inf_L	1,199
	L_parahippocampal	Fusiform_L	1,082
		Temporal_Pole_Sup_L	3,273
		Temporal_Pole_Mid_L	2,332
		Temporal_Inf_L	2,950
	R_entorhinal	Frontal_Inf_Tri_L	750
		Insula_L	2,504
		Cingulum_Ant_L	622
		ParaHippocampal_L	913
		Temporal_Pole_Sup_L	3,257
		Temporal_Pole_Mid_L	2,546
		Temporal_Inf_L	1,239
	R_parahippocampal	ParaHippocampal_L	896
		Fusiform_L	1,556
		Temporal_Sup_L	907
		Temporal_Pole_Sup_L	4,568
		Temporal_Pole_Mid_L	2,317
		Temporal_Inf_L	2,345
LMCI<CN	L_parahippocampal	Cingulum_Mid_R	274.00
		Cingulum_Post_R	357.00
		Cuneus_R	441.00
		Occipital_Sup_R	157.00
		Postcentral_R	475.00
		Parietal_Sup_R	1890.00
		Precuneus_R	2303.00
	R_isthmuscingulate	Cingulum_Mid_R	509.00
		Cingulum_Post_R	264.00
		ParaHippocampal_R	212.00
		Precuneus_R	824.00
LMCI>CN	R_isthmuscingulate	Cingulum_Mid_R	922.00
		Cingulum_Post_R	115.00
	R_posteriorcingulate	Cingulum_Ant_L	652.00
		Cingulum_Mid_L	328.00
		Cingulum_Mid_R	305.00

The proportion is the ratio of the voxel number with changed covariance in the atlas-based region to the total number of that region.

In Table 3, two attenuated covariant networks are identified in AD: (1) the correlation between left entorhinal and its covaried regions including bilateral posterior cingulate cortex and right precuneus. (2) The correlation between right entorhinal and left posterior cingulate cortex. These regions covered subnetwork of DMN, indicating a significant decrease in the covariant relationship between entorhinal and DMN subnetwork. In LMCI, two decreased covariant correlations were observed: (1) the connection between the DMN subnetwork (right middle and posterior cingulate cortex, and precuneus) and two seeds including left parahippocampal and right isthmus cingulate cortex. (2) The connection between right postcentral cortex and left parahippocaampal cortex.

On the other hand, the enhanced covariant networks in Table 3 reveal synchronous cortical changes between seeds and their associated regions. With all seeds showing significant CT thinning, it is expected that the corresponding covariant regions also exhibit a trend of cortical thinning. For instance, the left insula shows a trend of CT thinning in AD due to its covariation with five seeds. Also, two regions with significant trend of CT thinning in LMCI include bilateral middle cingulate and left anterior cingulate.

### 3.3. The functional networks

Using the rsfMRI data, functional connective networks and group ICA networks were estimated by CONN.

#### 3.3.1. Functional connective networks

According to the method in Section 2.5.4, after comparing the FC map of each seed between AD/LMCI and CN, the regions exhibiting significant group differences were identified as the inter-regional connective network. Table 4 summarize all inter-regional FC networks capable of distinguishing AD and LMCI from CN.

**Table 4:** The altered inter-regional functional connectivity in AD and LMCI.

Contrast	AAL-atlas-based seeds	Regions of changed functional connectivity	Number of verxel
AD<CN	Fusiform_L	Postcentral_L	178
		Juxtapositional Lobule Cortex	99
		Precentral_R	93
		Precentral_L	64
		Parietal_Sup_Lobule_L	43
		Cingulum_post	39
		Hippocampus l	32
		Precuneous Cortex	23
	Temporal_Pole_Sup_L	Precuneous Cortex	191
		Parietal_Sup_Lobule_R	137
		Occipital_Sup_R	67
	Temporal_Pole_Mid_L	Frontal Pole R	68
		Lingual Gyrus R	63
		Cingulate_Ant	59
		Paracingulate Gyrus R	53
		Lingual Gyrus L	23
	Fusiform_R	Frontal Pole R	79
		Insular Cortex R	25
AD>CN	ParaHippocampal_L	Frontal Pole Right	104
		Frontal_Sup_R	77
		Frontal_Sup_L	34
	Temporal_Pole_Sup_L	Lateral Occipital Cortex_R	269
		Precuneous Cortex	56
		Frontal Pole L	42
		Cingulum_Ant	33
		Paracingulate Gyrus Left	27
	Temporal_Pole_Mid_L	Cingulum_Ant	41
		Parietal_Operculum_R	12
	Temporal_Inf_L	Occipital_Sup_R	74
		Angular Gyrus R	69
		Superior Parietal Lobule R	22
LMCI>CN	Cingulum_Mid_R	Precuneous	221
		Paracingulate Gyrus R	117
		Superior Frontal Gyrus R	113
		Lateral Occipital Cortex L	35

Only the negative contrast identified FC networks which was not considered in our study.

Three inter-regional FC networks, located in the DMN (Precuneous Cortex, posterior cingulate cortex), sensorimotor network (bilateral precentral gyrus and left postcentral gyrus) and salience network (SN) (anterior cingulate, right paracingulate cortex and insular cortex), were found as indicated in Table 4 in bold. Compared with CN, the connectivity between these three networks and seeds was less significantly weakened (*p* < 0.05) in AD. For LMCI compared with controls, no significant reduction in FC correlative network was observed. Of note, enhanced FC networks were also found in AD and LMCI, which were not considered in this study.

#### 3.3.2. Group-ICA networks

Group ICA networks were generated for 40 ICs as described in Section 2.5.4. The slices of all 40 ICs in AD vs. CN and LMCI vs. CN are shown in Supplementary Figures S2, S3, respectively.

**Figure 3:**
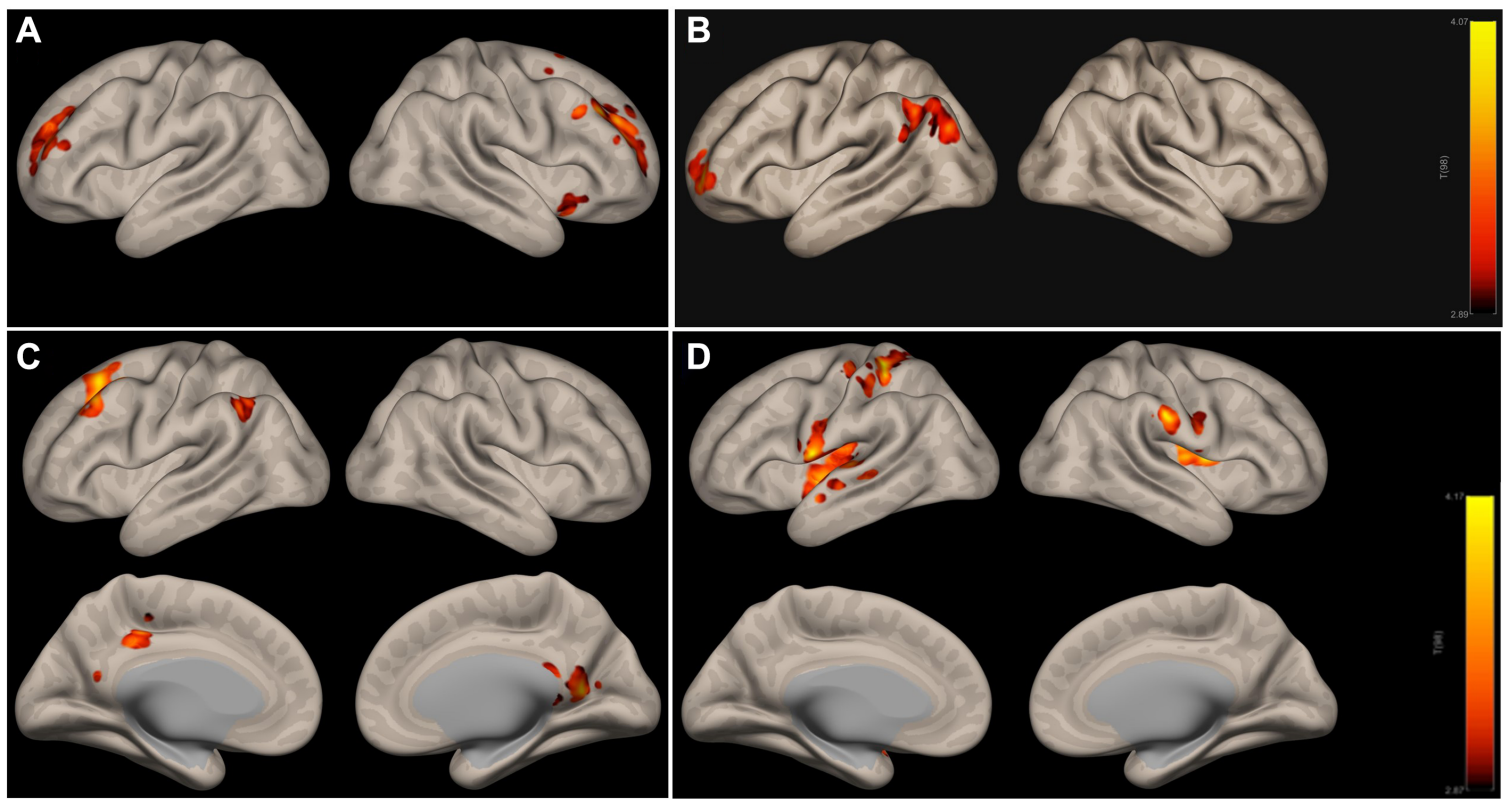
ICA networks with significant differences in AD **(A,B)** and LMCI **(C,D)** relative to CN. **(A,B)** Are subnetworks of DMN and SN; **(C,D)** are subnetworks of DMN and the motor network. The colorbar indicates the strength of IC weights, red indicates the strongest weight, and the color above red indicates the weaker weight. DMN, the default mode network; SN, salience network.

Nine out of the 40 ICs showed significantly decreased weights in AD compared to CN, as displayed in Supplementary Table S3. Figures 3A,B below illustrate two examples, IC16 and IC32, which correspond to DMN’s subnetwork (precuneous cortex, posterior cingulate gyrus) and SN (anterior cingulate gyrus, paracingulate gyrus, and insular cortex), respectively. In addition, IC3 as shown in Supplementary Table S3 corresponding to sensorimotor network, including precentral and postcentral gyrus, was also significantly different between AD and CN.

ICA networks in LMCI compared to CN are presented in Supplementary Table S4 and Figures 3C,D illustrate two examples, IC8 and IC22, which correspond to DMN’s subnetwork (precuneous cortex, posterior cingulate gyrus, left angular gyrus) and sensorimotor network (bilateral postcentral gyrus, left precentral gyrus, bilateral insular cortex), respectively.

#### 3.3.3. The consistent inter-regional networks

We examined the consistence between structural covariance, functional connectivity, and ICA networks to identify consistent inter-regional connectivity patterns.

According to Tables 3, 4 and Supplementary Table S3, for AD comparing with CN, the subnetwork of DMN, which includes posterior cingulate gyrus and precuneous cortex, is the consistent inter-regional connective network of the above three networks, while a subnetwork of SN (anterior cingulate gyrus, bilateral paracingulate gyrus and frontal) and a subnetwork of sensorimotor network (bilateral precentral gyrus and right postcentral gyrus) are contained in both FC network and ICA network, but not in covariance network.

For LMCI comparing with CN, as indicated in Tables 3, 4 and Supplementary Table S4, no significant reduced functional connective network was found, while a subnetwork of DMN (posterior cingulate gyrus and precuneous) and the motor areas (right postcentral and precentral cortex), consistently presented in both structural covariance network and group-ICA network.

## 4. Discussion and conclusion

In this study, we integrated three approaches – structural covariance, functional connectivity, and group ICA – to identify inter-regional networks that distinguish AD/LMCI patients from controls. First, we separately derived patient-specific networks using each method: seed-based structural covariance, seed-based functional connectivity, and group-ICA approach. Then, we extracted regions with consistent connectivity differences across these three network types in patients compared to controls. This allowed us to generate robust inter-regional networks specifically disrupted in AD and LMCI.

Brain atrophy in AD examined on sMRI by manual or automatic technique has been extensively used for early prediction of AD, but it still exhibits low sensitivity and specificity (Vemuri and Jack, 2010; Pini et al., 2016; Lombardi et al., 2020). Compared to GMV, cortical thickness provides an index to neuro number and density in a specific area and is widely utilized for measuring cortical atrophy (Fischl and Dale, 2000; Cheng et al., 2015), detecting disease progression (Phillips et al., 2019), and early prediction (Canu et al., 2017) in AD. In this study, the CT analysis was employed using voxel-wise GLM and the cortical atrophied regions with CT reduction were identified in AD and LMCI. Our results of cortical thinning in AD are consistent with previous studies (Phillips et al., 2019; Putcha et al., 2019). However, LMCI exhibited less pronounced cortical atrophy only in left entorhinal and parahippocampal cortex. Compared to a previous study (Canu et al., 2017), these biased results may have been caused by the different clinical populations, MRI scanners and statistical methods.

It is now widely accepted that AD damage results from disrupted connectivity between brain regions (Nestor et al., 2017; Park et al., 2017; Grajski and Bressler, 2019; Neitzel et al., 2019; Putcha et al., 2019; Berron et al., 2020; Tetreault et al., 2020) rather than individual atrophied region. Numerous studies on structural covariance (Abela et al., 2015; Valk et al., 2017) and functional connectivity (Kuang et al., 2019; Berron et al., 2020; Smith et al., 2021) have been conducted by pre-defining ROI seeds based on prior knowledge. Although the ROI-based approach is more reliable (Smitha et al., 2017), these predefined seeds ignore abnormal changes outside the defined ROIs and fail to reflect alterations in particular dataset. Contrastingly, we defined cortical atrophy regions with the CT thinning as the automatic and data-driven seeds for inter-regional correlation analysis, including cortical covariance and functional connectivity. This facilitate clear interpretation of brain atrophy as disruption of between-region connectivity. Additionally, group-ICA has been utilized for identifying distinct functional networks in a variety of disease (Caspers et al., 2021; Geng et al., 2022). In the study, group-level ICA networks were identified in AD or LMCI compared to CN groups.

The results in Tables 3, 4 and Supplementary Tables S3, S4 indicate that for AD, a subnetwork including posterior cingulate and precuneous cortex exhibits reduced connective correlation in both structural covariance and functional connectivity as well as reduced IC weights. The similar reductions in connectivity are observed in structural covariance and group ICA network for LMCI. Interestingly, group ICA consistently identifies the DMN subnetwork in AD, and sensorimotor network in both AD and LMCI, which were not necessarily fully identified by structural covariance and functional connectivity. This suggests that group ICA holds promise in replacing functional connectivity and structural covariance correlation to investigate brain network to some degree. Moreover, some increased connectivity correlations, that were identified by our method, have previously been detected in amyloid-β + MCI patients (Berron et al., 2020) and aging (Yao et al., 2013) which were more random. These enhanced connective correlation may stem from compensation mechanism to maintain memory, but the interpretation of the mechanism remains challenging and therefore was not considered in our current study.

This study has several limitations that could be addressed in future work: (1) The ICA performance depends on parameter selection. The number of ICs was fixed at 40 based on default settings, but robustness could be improved by testing a range of 30–100 ICs. (2) Only clinical diagnoses were used to construct the GLM model. Incorporating PET biomarkers, such as levels of tau accumulation, could enhance prediction accuracy of morphological damage concerning AD pathology. (3) Subcortical regions and DTI-based structural connectivity were not yet considered. Adding mesoscale subcortical networks and white matter connectivity could provide a more comprehensive connectomic characterization of AD. (4) We only recruited the late MCI subjects in this study. Adding early MCI could help enable earlier prediction of AD progression. (5) The diagnostic performance needs validation. Studies have shown that the classification performance by combining statistically significant morphological and functional information was significantly improved (Park et al., 2017; Li et al., 2020; Zhang et al., 2022). Therefore, using classification algorithms and related evaluation metrics to evaluate the diagnostic performance of our inter-regional connectivity network will be the focus of our next work.

In summary, we used structural covariance, functional connectivity, and group ICA networks to identify disrupted inter-regional connectivity in AD and LMCI. Our results suggest that patients with AD and LMCI exhibit reduced inter-regional connectivity within subnetworks of typical brain networks, such as DMN. These differences in inter-regional connectivity distinguish AD/LMCI patients from controls and provide insights into network-level disruptions underlying the disease. Overall, our multi-modal connectivity analysis may elucidate mechanisms of cognitive decline and identify imaging markers for diagnosis of AD/LMCI.

## Data availability statement

Publicly available datasets were analyzed in this study. This data can be found at: https://ida.loni.usc.edu/home/projectPage.jsp?project=ADNI.

## Author contributions

XR: Conceptualization, Data curation, Investigation, Methodology, Software, Writing – original draft, Writing – review & editing. BD: Data curation, Software, Writing – review & editing. YL: Data curation, Methodology, Writing – review & editing. YW: Methodology, Project administration, Supervision, Writing – review & editing, Conceptualization. YH: Conceptualization, Investigation, Methodology, Supervision, Writing – review & editing, Project administration.

## Alzheimer's Disease Neuroimaging Initiative

Data used in the preparation of this article were obtained from the Alzheimer’s Disease Neuroimaging Initiative (ADNI) database (adni.loni.usc.edu). As such, the investigators within the ADNI contributed to the design and implementation of ADNI and/or provided data but did not participate in the analysis or writing of this report. A complete listing of ADNI investigators can be found at: http://adni.loni.usc.edu/wp-content/uploads/how_to_apply/ADNI_Acknowledgement_List.pdf.

## Footnotes

^1^ http://adni.loni.usc.edu

^2^ https://www.math.mcgill.ca/keith/surfstat/

## Supplementary material

The Supplementary material for this article can be found online at:

https://www.frontiersin.org/articles/10.3389/fnhum.2023.1276994/full#supplementary-material
