# Corrigendum: Alterations via inter-regional connective relationships in Alzheimer's disease

**DOI:** 10.3389/fnhum.2023.1339574

**Published:** 2023-12-01

**Authors:** Xiaomei Ren, Bowen Dong, Ying Luan, Ye Wu, Yunzhi Huang, the Alzheimer's Disease Neuroimaging Initiative

**Affiliations:** ^1^College of Electrical Engineering, Sichuan University, Chengdu, China; ^2^Department of Radiology, Zhongda Hospital, School of Medicine, Southeast University, Nanjing, China; ^3^School of Computer Science and Engineering, Nanjing University of Science and Technology, Nanjing, China; ^4^Institute for AI in Medicine, School of Artificial Intelligence (School of Future Technology), Nanjing University of Information Science and Technology, Nanjing, China

**Keywords:** cortical thickness, cortical covariance network, vortex-wise general linear model, seed-based functional connectivity, group-level independent component analysis

An error in order was shown in the author list of our published article, and the correct author list should be as follows:

Xiaomei Ren^1†^, Bowen Dong^1†^, Ying Luan^2^, Ye Wu^3*^, Yunzhi Huang^4*^ and the Alzheimer's Disease Neuroimaging Initiative

The order of affiliations have also been corrected to account for the change in author order. It should be as follows:

^1^College of Electrical Engineering, Sichuan University, Chengdu, China

^2^Department of Radiology, Zhongda Hospital, School of Medicine, Southeast University, Nanjing, China

^3^School of Computer Science and Engineering, Nanjing University of Science and Technology, Nanjing, China

^4^Institute for AI in Medicine, School of Artificial Intelligence (School of Future Technology), Nanjing University of Information Science and Technology, Nanjing, China

The authors apologize for this error and state that this does not change the scientific conclusions of the article in any way. The original article has been updated.

