# Supplementary material for: Alterations via inter-regional connective relationships in Alzheimer’s disease
Source: Front Hum Neurosci. 2023 Nov 9;17:1276994. doi: 10.3389/fnhum.2023.1276994 (PMC10672243; doi:10.3389/fnhum.2023.1276994)
Supplement: Supplementary file 1 [file Data_Sheet_1.PDF]

Supplementary Material

Alterations in Alzheimer's Disease via Inter-Regional Covariance Networks

Xiaomei Ren[1†], Yunzhi Huang[†*2], Bowen Dong[1], Ying Luan[3], Ye Wu[*4]
For the Alzheimer's Disease Neuroimaging Initiative*[1]

[1] College of Electrical Engineering, Sichuan University, Chengdu, China

[2]Institute for AI in Medicine, School of Artificial Intelligence (School of Future Technology), Nanjing University of Information Science and Technology, Nanjing, China

[3]Department of Radiology, Zhongda Hospital, School of Medicine, Southeast University, Nanjing, China

[4]School of Computer science and engineering, Nanjing University of Science and Technology, Nanjing, China

***Correspondence:**
Yunzhi Huang
huang_yunzhi@nuist.edu.cn

Ye Wu
wuye@njust.edu.cn

† These authors contributed equally to this work and should be considered co-first authors.

Keywords: Cortical thickness; Cortical covariance network; Vortex-wise general linear model; Seed-based functional connectivity; Group-level independent component analysis

Supplementary Material 1. the Group-Level Analysis of Cortical Thickness

The vertex-wise GLM was conducted using SurfStat Toolbox[2] for CT analysis. The CT value at each vertex was modeled as the dependent variable, with group (CN, AD, LMCI) as the independent variable. Age, sex, education, scanner type, and total GMV were included as covariates. Cluster-level statistics were thresholded at p<0.001 for AD vs CN and p<0.025 for LMCI vs CN to correct for multiple comparisons using random field theory (RFT). A cluster extent threshold of p<0.05 family-wise error (FWE) rate

---

[1] *Data used in the preparation of this article were obtained from the Alzheimer's Disease Neuroimaging Initiative (ADNI) database (adni.loni.usc.edu). As such, the investigators within the ADNI contributed to the design and implementation of ADNI and/or provided data but did not participate in the analysis or writing of this report. A complete listing of ADNI investigators can be found at: http://adni.loni.usc.edu/wp-content/uploads/how_to_apply/ADNI_Acknowledgement_List.pdf

[2] https://www.math.mcgill.ca/keith/surfstat/

was then applied to control the probability of reporting false positive clusters. The cortical regions demonstrating significant group differences were mapped onto the Destrieux atlas to generate ROIs called seeds. These regions were also mapped onto the Anatomical Automatic Labeling (AAL) atlas in MNI space to create AAL-based seeds for FC analysis.

Multiple regions exhibited CT thinning in AD and LMCI patients compared to controls, but no cortical thickening was found in either. The relative results is shown in Fig. s1.

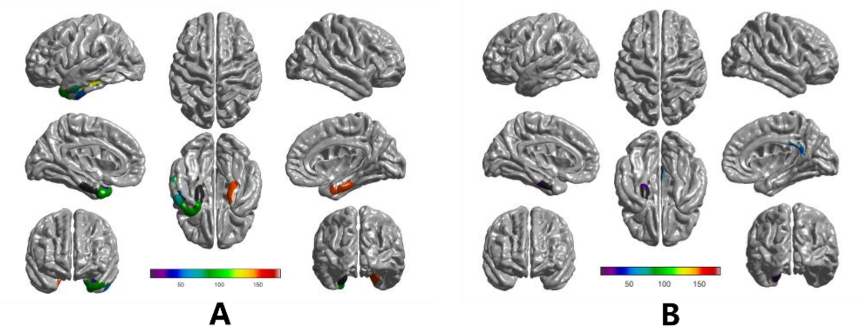

Fig. s1 Multiple regions exhibited CT thinning in AD (A) and LMCI (B) patients compared to controls. The different colors consistent with colorbar indicate that they are matched to different brain regions in the Destrieux atlas.

Table 2 lists the regions showing significant cortical thinning in AD/MCI vs controls after mapping to the Destrieux atlas. The cortical thinning regions in surface space were then converted into MNI volume space and localized using the AAL atlas to generate ROIs for further analysis, as shown in Table 2.

In addition, the results of thinning cortical regions in AD vs. LMCI were also assessed in this study, and the following Table s1 reports the cortical thinning regions in Destrieux and AAL atlas for AD campared with LMCI.

Table s1 The cortical thickness thinning regions in AD versus LMCI.

Destrieux atlas-based CT thinning regions			AAL atlas-based CT thinning regions		
Destrieux-atlas-based seeds	voxel number	the thinning ratio	AAL-atlas-based seeds	voxel number	the thinning ratio
L_bankssts	109	5.10	Temporal_Mid_L	1744	4.43
L_entorhinal	208	18.87	ParaHippocampal_L	1902	24.10
L_isthmuscingulate	569	22.48	Cingulum_Mid_L	398	2.57
L_medialorbitofrontal	616	23.22	Cingulum_Post_L	553	14.89
L_middletemporal	497	11.16	Frontal_Med_Orb_L	1016	17.54
L_parahippocampal	651	35.42	Rectus_L	540	7.87
L_precuneus	565	7.73	Precuneus_L	1503	5.30
L_supramarginal	423	4.92	SupraMarginal_L	1072	10.82
R_bankssts	413	18.81	Occipital_Mid_L	157	0.60
R_inferiorparietal	493	5.10	Fusiform_L	143	0.78
R_isthmuscingulate	169	7.08	Temporal_Inf_L	534	2.08

R_middletemporal	143	2.83	Parietal_Inf_R	1387	12.89
R_precuneus	174	2.18	Cingulum_Mid_R	120	0.69
R_supramarginal	129	1.58	Cingulum_Post_R	159	5.99
			Precuneus_R	555	2.13
			Temporal_Mid_R	1290	3.64

Supplementary Material 2. Seed-based Structural Covariance Analysis

An interactive GLM approach was used to examine whether a specified seed region exhibited distinct cortical covariance patterns across the entire surface in patients compared to controls. Mathematically, the interactive GLM can be formulated as follows:

$$\boldsymbol{CT_i = (\beta_{i0} + \beta_{i1} * Sex + \beta_{i2} * Age + \beta_{i3} * Diagnosis + \beta_{i4} * Education + \beta_{i5} * Scanner + \beta_{i6} * GMV) * (1 + \beta_{i7} * CT_{seed})}, \quad (1)$$

where * denotes an interaction, the weight coefficients of each covariates are denoted by $\boldsymbol{\beta_i}$, CT_i represents the CT data of the *i*th voxel, and *CTseed* is the average CT of a specific seed, GMV refers to total GMV, Diagnosis is the group identifier. The contrast for comparing AD and CN can be: *contrast* = *CTseed.*Diagnosis.CN-CTseed.*Diagnosis.AD*. The results were corrected using RFT at a cluster threshold of p=0.025, controlling probability for FWE at cluster-wise threshold $PFWE$<0.05. All surviving regions after correction were matched onto AAL atlas.

Table 3 summarizes the altered cortical covariant networks in AD and LMCI compared to CN. And the same result with AD versus LMCI is shown in following Table s2.

Table s2 the cortical covariant regions in patients with AD relative to LMCI

contrast	AAL-atlas-based seeds	Regions of changed functional connectivity	number of verxel
LMCI>AD	'Cingulum_Post_L	Lateral Occipital Cortex, superior division R	99

Supplementary Material 3. Group-level ICA.

Group-ICA [1] were performed to estimate 40 temporally coherent networks from the rsfMRI data across all subjects. The BOLD time series from every voxel in the brain across all subjects were temporally concatenated. A singular value decomposition (SVD) of the z-score normalized BOLD signal with 64 components separately for each subject was used as a subject-specific dimensionality reduction step. The dimensionality was further reduced to 40 components with SVD, and a fast-ICA fixed-point algorithm [2] was used to identify spatially independent group-level networks from the resulting components. Lastly, a back-projection [3] was used to compute ICA networks associated with these same networks separately for each subject. Group ICA networks were generated by the group-level analysis for 40 ICs.

The slices of all 40 ICs were shown in Figure s2 and s3 bellow, respectively. Nine ICs

out of the 40 ICs showed significantly decreased IC weights in AD as compared with CN, as displayed in Table s3; ICA networks for LMCI compared with CN are presented in Table s4.

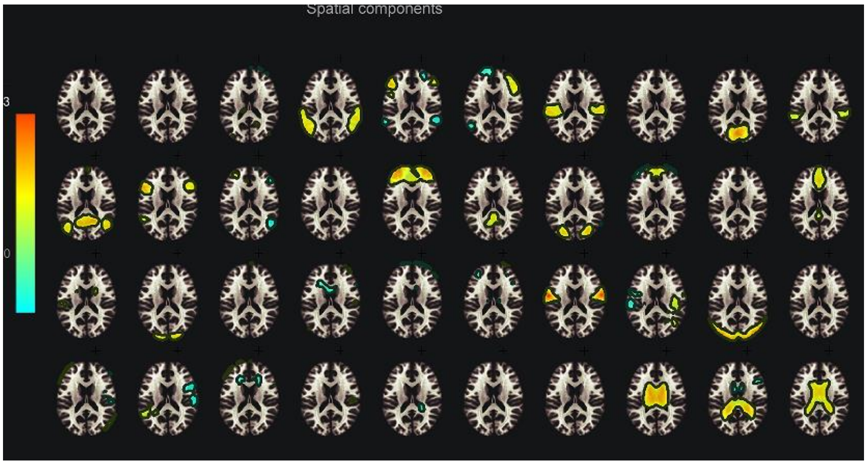

Fig s2 All 40 ICs from the group-ICA networks for AD comparing with CN.

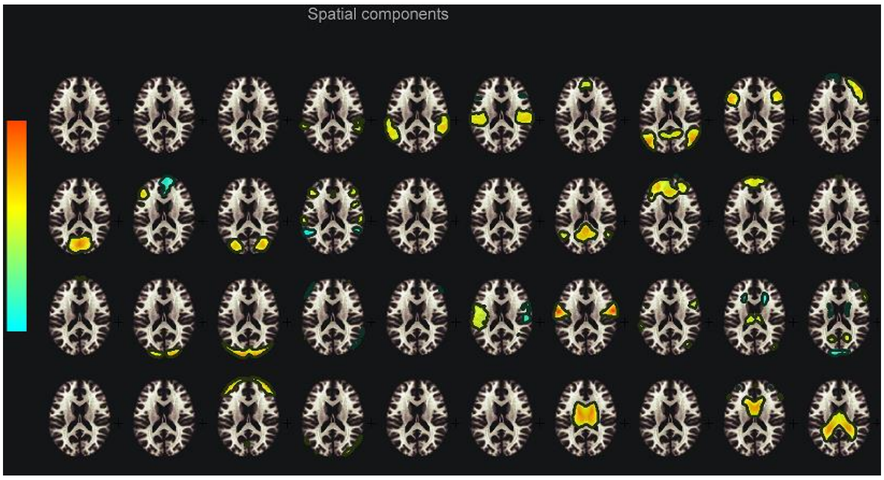

Fig s3 All 40 ICs from the group-ICA networks for LMCI comparing with CN.

Table s3 The significant ICA networks with AD compared to CN

IC number	contrast : CN>AD	The voxel sum
1	Frontal Pole R	323
	Frontal Pole L	226
	Frontal Orbital Cortex L	179
	Frontal Orbital Cortex R	93
3	Postcentral Gyrus L	398
	Superior Frontal Gyrus R	372
	Superior Frontal Gyrus L	221
	Precentral Gyrus L	72
	Precentral Gyrus R	31
5	Inferior Temporal Gyrus, temporooccipital part L	299
	Inferior Temporal Gyrus, posterior division L	231
	Lateral Occipital Cortex, superior division L	165

	Temporal Fusiform Cortex, posterior division L	66
	Middle Temporal Gyrus, temporooccipital part L	40
	Temporal Occipital Fusiform Cortex L	32
6	Supramarginal Gyrus, posterior division R	135
	Angular Gyrus R	66
	Middle Temporal Gyrus, posterior division R	49
	Inferior Temporal Gyrus, posterior division R	39
11	Lateral Occipital Cortex, superior division L	565
	Lateral Occipital Cortex, superior division R	398
	Superior Frontal Gyrus L	166
	Frontal Pole L	107
	MedFC (Frontal Medial Cortex)	87
	Frontal Pole R	61
16	Cingulate Gyrus, anterior division	747
	Frontal Pole L	653
	Frontal Pole R	616
	Paracingulate Gyrus R	484
	Paracingulate Gyrus L	320
	Middle Frontal Gyrus R	194
	Insular Cortex R	114
	Frontal Orbital Cortex R	87
20	Supramarginal Gyrus, anterior division R	200
	Frontal Pole R	141
	Middle Temporal G	116
	Lateral Occipital Cortex, inferior division L	115
	Middle Temporal Gyrus, temporooccipital part L	61
	Planum Temporale R	56
	Supramarginal Gyrus, posterior division R	54
	Supramarginal Gyrus, anterior division L	35
	Lateral Occipital Cortex, inferior division R	35
	Superior Temporal Gyrus, posterior division R	27
	Supramarginal Gyrus, posterior division L	23
32	Precuneous Cortex	269
	Lateral Occipital Cortex, superior division L	258
	Cingulate Gyrus, posterior division	237
	Frontal Pole L	210
	Angular Gyrus L	103
35	Supramarginal Gyrus, posterior division L	200
	Middle Temporal Gyrus, posterior division R	181
	Angular Gyrus L	106
	Inferior Temporal Gyrus, posterior division R	43

Table s4 The significant ICA networks with LMCI compared to CN

IC number	contrast : CN>LMCI	the voxel number
8	Middle Frontal Gyrus L	439
	Superior Frontal Gyrus L	313
	Precuneous Cortex	175
	Cingulate Gyrus, posterior division	95
	Angular Gyrus L	89
	Supramarginal Gyrus, posterior division L	85
12	Inferior Temporal Gyrus, temporooccipital part L	312
	Superior Frontal Gyrus L	129
	Middle Frontal Gyrus L	110
	Inferior Temporal Gyrus, posterior division L	105
	Temporal Fusiform Cortex, posterior division L	22
22	Postcentral Gyrus L	266
	Central Opercular Cortex L	245
	Insular Cortex L	180
	Precentral Gyrus L	164
	Insular Cortex R	154
	Postcentral Gyrus R	51

References

1. Calhoun, V.D., Adali, T., Pearlson, G. D., & Pekar, J. J., *A method for making group inferences from functional MRI data using independent component analysis.* Human brain mapping, 2001. **14**(3): p. 140-151.
2. Hyvarinen, A., *Fast and Robust Fixed-Point Algorithms for Independent Component Analysis.* IEEE Transactions on Neural Network, 1999. **10**(3): p. 626-634.
3. Erhardt, E.B., Rachakonda, S., Bedrick, E. J., Allen, E. A., Adali, T., & Calhoun, V. D., *Comparison of multi‐subject ICA methods for analysis of fMRI data.* Human brain mapping, 2011. **32**(12): p. 2075-2095
